# Evaluation of Optimal Insertion Sites and Angles for Orthodontic Mini-Implants at the Anterior Nasal Spine Region Based on Cone-Beam Computed Tomography

**DOI:** 10.3390/jcm13030837

**Published:** 2024-02-01

**Authors:** Donger Lin, Shangyou Wen, Zelin Ye, Yi Yang, Xuechun Yuan, Wenli Lai, Meng You, Hu Long

**Affiliations:** 1 Department of Orthodontics, State Key Laboratory of Oral Diseases, National Center for Stomatology, National Clinical Research Center for Oral Diseases, West China Hospital of Stomatology, Sichuan University, Chengdu 610041, China; icegooselin@gmail.com (D.L.); aw9481@gmail.com (S.W.); yy18583233717@163.com (Y.Y.); yuanxc@foxmail.com (X.Y.); wenlilai@scu.edu.cn (W.L.); 2 Department of Oral Radiology, State Key Laboratory of Oral Diseases, National Center for Stomatology, National Clinical Research Center for Oral Diseases, West China Hospital of Stomatology, Sichuan University, Chengdu 610041, China; yeahzelin@gmail.com (Z.Y.); youmeng@scu.edu.cn (M.Y.)

**Keywords:** orthodontics mini-implant, anterior nasal spine, cone-beam computed tomography

## Abstract

Background: To infer the optimal angulation and height level of mini-implant insertion in the anterior nasal spine (ANS) region from alveolar bone depth (BD) and labial cortical bone thickness (CBT) through cone-beam computed tomography (CBCT), the influences of sex, age, and growth pattern on BD, CBT, and the starting point of the ANS were examined. Material and methods: BD and CBT were measured on CBCT median sagittal images at specific angles (0, 15, 30, 45, and 60 degrees) to simulate the angulation of insertion. The height level of the first axial image with obvious bone ridge was recorded as the start of ANS. Results: The average height of ANS start was 9.42 mm from the alveolar bone crest between the central incisors. The variations in height level and insertion angle combined, or either of them individually, significantly influenced BD and CBT, demonstrating overall decreasing trends (*p* < 0.001). BD was not influenced by sex, age, or growth pattern (*p* > 0.05). However, CBT was found to be correlated with sex and growth pattern, while the height level of ANS start was associated only with growth pattern, particularly in hypodivergent patients (*p* < 0.05). Conclusion: The recommended insertion height level is 14–16 mm from the alveolar bone crest and the recommended angulation is 30–45°. An innovative inequity in the relationship between a certain height level and the insertion angulation is proposed.

## 1. Introduction

Mini-implants have recently aroused growing attention in orthodontic therapy. They act as temporary anchorage devices, playing crucial roles in torque control and vertical movement during clear aligner therapy. Specifically, when placed in the anterior maxilla, they are proven to provide valuable anchorage for clinicians in addressing excessive gingival display via anterior teeth intrusion and severe deep bite cases that require effective torque control [1,2,3]. Notably, the use of mini-implants in these situations has demonstrated reduced anchorage loss compared to conventional approaches, such as reverse curve archwires and other extraoral anchorage devices [3].

In the past, the inter-radicular space between the central incisors was the most common site for mini-implant placement, enabling simultaneous proclined and intrusive effects on maxillary anterior teeth. Nevertheless, a considerable proportion of malocclusion patients exhibit anterior teeth crowding [4,5,6], resulting in a restricted inter-radicular space between the central incisors that is insufficient to accommodate a 1.5–2 mm diameter mini-implant. Additionally, the use of a thinner diameter mini-implant for anchorage may not provide adequate stability [7]. The tooth root–mini-implant contact may give rise to detrimental consequences, such as root damage, and thus ultimately results in the loosening and dislodgement of the mini-implant. One potential approach involves augmenting the angulation and insertion height level of the mini-implant to attain safer distance between roots.

The anterior nasal spine (ANS) region emerges as a recommended alternative as it offers a comparable force application pattern and possesses adequate bone volume devoid of root structures [8,9]. The ANS is a small protruded bone above the alveolar bone, located in the midline of the maxilla and at the bottom of the nose and lower edge of the piriform aperture [10]. Empirical execution of mini-implant insertion has been prevalent, but no research has been conducted to investigate the optimal insertion height and angulation. The failure of mini-implant insertion has been reported to be influenced by both the anatomical conditions of the insertion site and the mechanical parameters of the mini-implant [11]. Miyawaki et al. [12] indicated that the stability of mini-implants was affected by the thickness of the cortical bone and the configuration of the titanium mini-implant. The stability of mini-implant insertion is largely affected by age, sex, craniofacial skeletal patterns, angulation to bone, insertion torque, quality and quantity of the cortical bone, thickness and mobility of the soft tissue, the dimension and configuration of the titanium mini-implant, and root proximity [13,14,15,16,17,18,19]. Motoyoshi M et al. [20] proposed that cortical bone thickness is the key factor influencing the success rate of mini-implants. Due to the special anatomic configuration of the ANS region, BD and CBT may vary according to height and angulation. Therefore, our research shed lights on mapping the variations in alveolar bone depth (BD) and labial cortical bone thickness (CBT) in the ANS region with respect to height and angulation in the hope of establishing guidance and instruction for clinicians regarding the optimal height and angulation.

This study employed cone-beam computed tomography (CBCT) scans to measure various anatomical parameters, including alveolar bone depth (BD) and labial cortical bone thickness (CBT) in the ANS region. Sex, age, and growth pattern were examined to reveal their influences on BD and CBT. The aim of this research was to deduce the optimal insertion angulation and height for mini-implants in the ANS region.

## 2. Materials and Methods

### 2.1. Samples

A total of 50 Chinese orthodontic patients were included in this study. The sample size was calculated with a confidence of 95%%, (α = 0.05), a sample power of 90%, and a moderate effect size (0.25), using GPower software version 3.1.9.4. [21]. To account for possible exclusions, a total of 50 participants were selected.

The CBCT images were uniformly obtained and scanned by the same radiologist. The inclusion criteria were: (1) completely erupted maxillary central incisors without malformation; (2) no maxillary skeletal defect; and (3) no history of medical interventions that may cause bone remodeling in the apical area of the maxillary central incisors and ANS region. The exclusion criteria included: (1) missing maxillary anterior teeth; (2) impacted maxillary anterior teeth; (3) maxillary skeletal defects; and (4) unclear or incomplete CBCT images. The study cohort was stratified into distinct groups based on participant sex, age, and growth pattern, facilitating subsequent analyses. According to the cephalometrics of the included subjects, “GoGn-SN” was utilized to differentiate between hyperdivergent, normodivergent, and hypodivergent (GoGn-SN: average: 32.5°, SD: 5.2°). Prior to the commencement of data collection, ethical considerations were upheld and informed consent was obtained from all participants or their legal guardians.

### 2.2. CBCT Examinations

The three-dimensional volume scanner adopted in this research was an MCT-1 CBCT scanner from J Morita Mfg Corp. in Kyoto, Japan. The scanner settings were as follows: 85 kV (anterior posterior–latero lateral), 5.0 mA (anterior posterior), and 5.0 mA (latero lateral); exposure time 17.5 s; and slice thickness 1.0 mm.

### 2.3. Measurement Protocol

**Figure 1:**
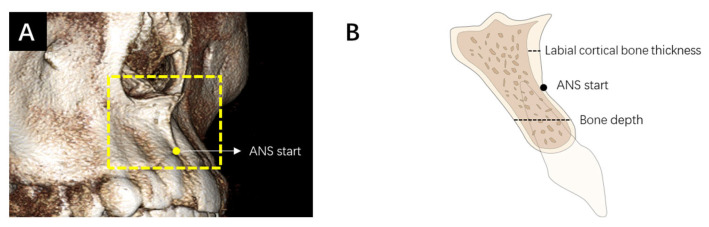
(**A**) Three-dimensional reconstruction of the ANS area. (**B**) Illustration of the median sagittal image showing the anatomical information.

The measurements of alveolar bone depth and cortical bone thickness were completed by the same orthodontist using Invivo6 software (Anatomage Inc., San Jose, CA, USA). Before starting the measurements, the occlusal plane was determined on the transverse plane by a line simultaneously passing the midpoint of occlusion of the bilateral first molars in the coronal plane and the midpoint of overbite in the sagittal plane (Figure 1). The measurements of alveolar BD and CBT were performed at the median sagittal plane. Firstly, a vertical line incorporating a 2 mm scale was drawn from the alveolar crest to the highest point of the ANS to represent its vertical height as well as provide a reference for the beginning and ending points of the whole measurement. Each 2 mm increment in vertical height would require a repeated measurement of alveolar BD and CBT at the corresponding height level. The 2 mm interval was determined according to previous studies [22,23]. The measurement lines originated from the labial edge of the alveolar bone at various height levels and extended to the palatal edge of the alveolar bone or the labial cortical bone, representing the measurements of alveolar BD and CBT, respectively. All of the measurement lines were parallel to each other and remained horizontal, reflecting the direction of mini-implant insertion (Figure 1). Furthermore, in order to simulate the different angles of mini-implant insertion, the CBCT images were rotated counterclockwise at 15°, 30°, 45°, and 60° with respect to the occlusal plane, respectively. The adjacent lines with an interval of 2 mm remained parallel. The measurement protocol was repeated for each rotation angle.

The identification of the starting point of the ANS (ANS start) was performed on the axial plane, and the corresponding vertical height level was documented. To assess the measurement consistency, a subset of 20% of the samples was randomly selected 2 weeks after the initial measurements and remeasured by the same orthodontist as an intra-examiner reliability evaluation.

### 2.4. Statistical Analysis

Statistical analysis was performed using Graphpad Prism, Version 9 (GraphPad Software Inc., San Diego, CA, USA) and Statistical Package for the Social Sciences software, Version 22.0 (SPSS Inc., Chicago, IL, USA). All data at different height levels and insertion angles were assessed by two-way analysis of variance. The height of ANS start was assessed using the *t*-test for independent samples. The level of significance was set at α = 0.05. Meanwhile, linear regression analysis was carried out in order to explore whether factors such as age, sex, and growth pattern would affect the variations in BD, CBT, and the height level of ANS start. The consistency test was indicated by the intraclass correlation coefficient value (ICC). If the ICC value exceeded 0.75, the consistency would be regarded as good.

## 3. Results

### 3.1. Patients Profile and Consistency

The study cohort consisted of CBCT images from 50 Chinese patients, ranging from 12 to 32 years old (mean age: 22 years old). Among them, 54% were female and 46% were male. Normodivergent patients accounted for the highest proportion (42%), while the proportion of hypodivergent patients was the second highest (34%), and hyperdivergent patients came third (24%). The ICC value was 0.845 > 0.75, indicating a high level of agreement of consistency (Table 1).

### 3.2. Determine ANS Start

**Figure 2:**
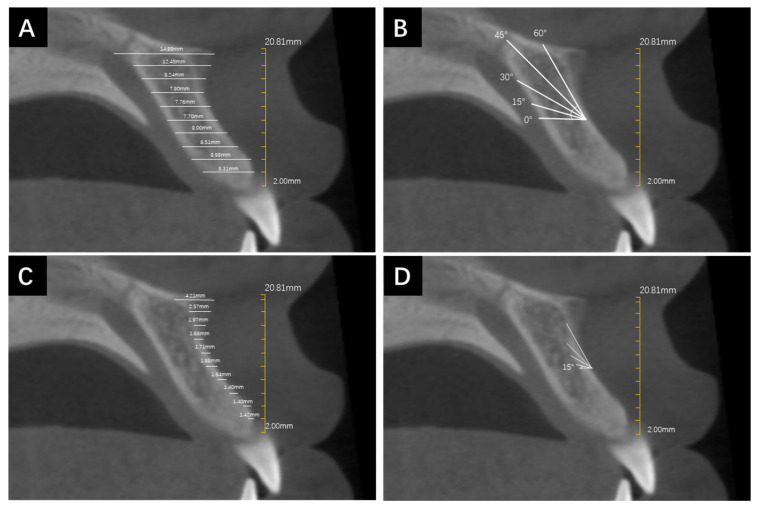
The measurement method of bone depth and cortical bone thickness in different rotation angles. (**A**) The measurement lines originated from the labial edge of the alveolar bone at various height levels and extended to the palatal edge of the alveolar bone to represent alveolar BD. (**B**) CBCT image rotated counterclockwise 15°, 30°, 45°, and 60° to simulate BD measurements under different insertion angles. (**C**) The measurement lines originated from the labial edge of the alveolar bone at various height levels and extended to the edge of labial cortical bone to represent CBT. (**D**) CBCT image rotated counterclockwise 15°, 30°, 45°, and 60° to simulate CBT measurements under different insertion angles.

**Figure 3:**
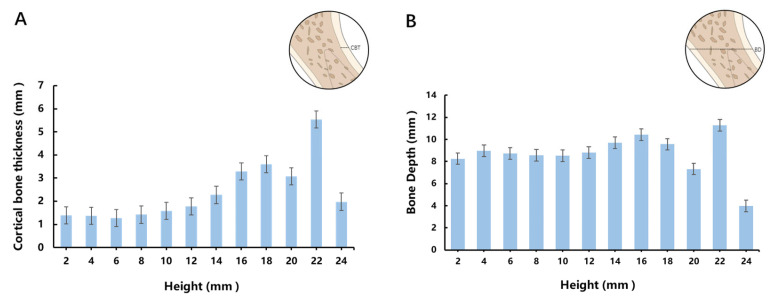
(**A**) The variation trend in cortical bone thickness at different height levels. (**B**) The variation trend in bone depth at different height levels.

Based on the measurements, the average height of ANS start was 9.42 mm from the alveolar bone crest between the central incisors. Therefore, we set the baseline at 10 mm on the x-axis to represent the ANS start, as displayed in Figure 2 and Figure 3.

### 3.3. The Relationship between CBT and BD with Respect to Height Variations at 0°, 15°, 30°, 45°, and 60° Insertion Angles

Figure 2 illustrated the trends in CBT and BD variations with respect to height. The CBT curve in Figure 2A showed an initial increase followed by subsequent decrease, with 22 mm as its peak and 24 mm as its trough. As for the BD curve in Figure 2B, there was a uniform BD of 8–9 mm before the 18 mm height level, followed by a sudden decrease at 20 mm, with a peak at 22 mm and minimum at 24 mm. The mixed effects analysis revealed that the value of ANS CBT and BD were affected by either the height or insertion angle individually or by both factors simultaneously (Table 2).

In Figure 3A, the mixed effects analysis showed that for insertion angles of 0°and 15°, BD initially demonstrated a slight increase within the height range of 10 mm to 14 mm. However, when the height level was higher than 14 mm, BD tended to decrease. Notably, when the insertion angle was 0° and the height reached 22 mm, BD showed a sharp increase. In contrast, for insertion angles of 30° and 45°, BD exhibited a consistent downward trend during the whole course. As for an insertion angle of 60°, BD remained lower than 5 mm when the height exceeded 14 mm. The area under the curves (AUC) in Figure 3B exhibited a steady decrease as the insertion angle increased and the one-way ANOVA analysis of AUC revealed a statistically significant difference (*p* < 0.05).

**Figure 4:**
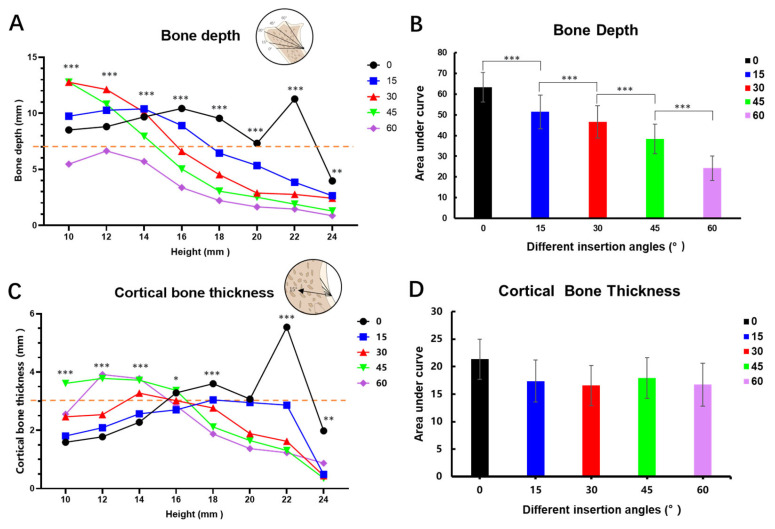
(**A**) Mixed effects analysis of bone depth in different height levels and insertion angles. (**B**) The area under the curves of graph (**A**). (**C**) Mixed effects analysis of cortical bone thickness at different height levels and insertion angles. (**D**) The area under the curves of graph (**C**). *: *p* < 0.05; **: *p* < 0.01; ***: *p* < 0.001.

Figure 4C illustrated the fluctuation of CBT with respect to varying height levels. The 0 and 15 degree curves mainly exhibited initial increases followed by sharp decreases, and the rest demonstrated a consistent downward trend, similar to Figure 3A. When the height exceeded 16 mm, all of the curves showed a downward trend, except for 0°. Conversely, when the height was less than 16 mm, most of the curves displayed an upward trend and gradually flattened. However, the AUC did not exhibit significant statistical differences across different insertion angles (Figure 4D).

### 3.4. The Relevance between Alveolar Bone Parameters and Sex, Age, and Growth Pattern

Linear regression analysis was adopted to reveal the relevance between the alveolar bone-related measurement results and variables such as sex, age, and growth pattern. As Table 3 demonstrates, there was no statistically significant association between BD and sex, age, and growth pattern (*p* > 0.05). However, CBT exhibited a statistically significant correlation with sex (β = 0.336, 95% CI 0.111–1.064; *p*-value < 0.05) and growth pattern (hypodivergent β = 0.301, 95% CI 0.026–1.083; *p* < 0.05), except for age. For the height level of ANS start, we also found that only growth pattern was significantly correlated, especially in the hypodivergent type (hypodivergent β = 0.0–0.370, 95% CI −2.239–−1.129; *p* < 0.001), while age and sex were not statistically relevant.

## 4. Discussion

The ANS connects with the alveolar bone to form a continuum, with its starting point being the most concave. The CBT curve in Figure 3A demonstrated first an upward trend and then a sharp decrease at 0°. At the 22 mm peak, most of the images were observed to manifest a continuous palatal plate full of cortical bone, as was indicated by 2 times the CBT value of the 20 mm height. You et al. [24] classified the morphology of the ANS region into four categories (double rod, single rod, triangular, and irregular) based on 1000 pieces of transverse CT samples, which is a perfect illustration of the anatomic variations in ANS morphology. The trend could be attributed to the anatomical variations in the ANS, such as in an irregular phenotype in which the loss of ANS prominence may result in a dramatic decrease at 24 mm. Figure 3B exhibited a uniform BD below 18 mm, while 20 mm showed the most concave ANS start, and 22 and 24 mm indicated untrustworthy results due to morphological variations in the hard palate and ANS, as explained above.

In this research, ANS start was defined as the beginning of the bone ridge, which was easily identifiable in CBCT axial images. Linear regression analysis (Table 3) indicated a significant association between the height of ANS start and growth pattern as well as sex. The septal-traction model describes the development of the ANS involving the septal cartilage, the downward and forward growth of which pulls the premaxilla to penetrate the fibrous perichondral attachment and forms a septopremaxillary ligament (SPL) at the site of the ANS. When the premaxillary-maxillary suture closes, SPL growth continues and renders the ANS more prominent and larger [25]. A study of American black and white specimens discovered that black specimens had a smaller or absent ANS, prognathic facial profile, and patent sutures, while white specimens exhibited a prominent ANS, orthognathic facial profile, and more fused sutures [26].

Our result is the first to reveal that the ANS start is lower and thus more occlusal in hypodivergent patients, suggesting its size is larger. The hypothesis can be raised that they exhibit more fused premaxillary-maxillary suture. Future study will discover the mechanism and validate the association between ANS start and facial profile. Therefore, it is safer for clinicians to insert mini-implant screws more occlusally around the ANS start to avoid perforation of the palatal plate while achieving a more satisfactory CBT as retention.

Anatomical considerations should be taken into account when planning mini-implant insertions to ensure optimal outcomes and avoid potential complications. The ANS region is in close proximity to the delicate nasal base and may lead to nasal base perforation during insertion. Additionally, cracks or fractures in the fragile bone ridge would compromise long-term stability. Notably, the nasopalatine canal traversing along the palatal alveolar bone is a potential site of injury in cases of severe maxilla atrophy and subsequent neurological damage can result in paresthesia.

Although there were instances [27] of intentionally utilizing the nasopalatine canal as an anatomical buttress for dental implant insertion and no presented paresthesia, mini-implants are different for their lack of osseointegration and solely rely on mechanical retention. Therefore, it is preferable for mini-implants to be inserted into the cortical bone with sufficient and even maximal length [28]. Besides actively avoiding these anatomic structures, ensuring adequate BD during insertion is of paramount importance. At a certain height level, a negative association between BD and insertion angle was observed. But no statistically significant correlation was found between BD and age, sex, and growth pattern. As a result, indiscriminately increasing the insertion angle in pursuit of more inter-radicular space may result in insufficient BD and thus pose risks of mini-implant failure [29]. According to Shouichi Miyawaki et al. [12], if the length of an intra-bony mini-implant was equal to or greater than 5 mm, stability was not correlated with increased length. Taking into account the necessary bone contact and bone trimming loss (2 mm) during insertion, it was observed that sites with a minimal 7 mm BD were suitable for accommodating mini-implants.

Our study revealed a significant correlation between CBT and BD with respect to height variations under different insertion angles. Increasing the insertion height and insertion angle are two viable solutions to minimize the risk of root damage and maximize inter-radicular space. There was an evident downward trend overall in Figure 4A. The shape of the 0 degree BD curve reflected the buccal morphology and anatomical variations in the alveolar–ANS complex, as elucidated above. However, at 30, 45, and 60 degrees, the line of insertion severed the continuum into triangle-like shapes, of which BD formed a base that was reduced at increased height levels and was even almost diminished at 22 mm and 24 mm as it reached the very narrow peak (ANS) of the triangle. The 15 degrees result may have demonstrated a trend more similar to the 0 degree result due to the minor change in angulation. The large inclination of the protrusion of the alveolar ridge may obstruct the placement of the insertion handpiece, and thus only height levels above the concavity (ANS) could be measured to exhibit BD. On the other hand, CBT exhibited no such morphology-related results due to it being mainly influenced by metabolism and mechanical factors [30].

These findings indicate that precautions, like reducing the length of the mini-implant, must be taken to avoid perforation of the palate. The deficiency in CBT can be compensated for by variations in mini-implant angulation or perhaps implant design (cylindrical vs. conical shank) [31].

BD is mainly influenced by the morphology of the ANS region and is not significantly associated with age, sex, and growth pattern. However, previous research has shown that in short face individuals, the BD of the anterior maxilla is higher than that in long or average face individuals [32].

To date, two anatomical factors are proven to influence the success rate of mini-implant insertion: root proximity and CBT [33]. Close root proximity poses potential risks to root damage and undesired mechanical interference. The average height of ANS start was measured to be 9.42 mm from the alveolar bone crest between the two central incisors. For a normal central incisor, the average length of its root ranges from 8.89 mm to 14.99 mm [34]. Consequently, in most patients, only the apical portion of the dental roots is present in the ANS region. Therefore, the apical inter-radicular space between these roots is larger than that between the middle part, rendering it more likely to accommodate mini-implants [35,36] without damaging the roots.

Previous research indicated that a minimum CBT of 1 mm was necessary to achieve primary stability [20]. Given the existence of a keen-edged bone ridge, bone trimming becomes necessary during mini-implant placement in the ANS region. An estimated 2 mm depth of bone removal is required to create an insertion platform and prevent implant slippage. Consequently, CBT should exceed 3 mm for adequate stability. Figure 4C demonstrated that when the CBT threshold was set at 3 mm, satisfactory CBT was predominantly centered around the height level of 14 mm to 16 mm, and qualified insertion angles included 30°, 45°, and 60°. Notably, considering the required BD threshold of 7 mm in Figure 3A, our findings revealed that insertion angles of 0° to 30° provided satisfactory BD across all height levels, concentrating at height levels ranging between 10 mm and 16 mm. Conversely, for an insertion angle of 60°, BD fell below 7 mm at all height levels. Combining Figure 4A,C, the range of suitable insertion angles was determined to be 30°–45°. Moreover, Letizia Perillo et al. [37] proposed in a finite element analysis study investigating mini-implant placement in the mandible that an insertion angle of 60° or 120° from the bone surface is susceptible to the greatest cortical bone stress, while this study suggested an insertion angle of 30° to the occlusal surface falls into this category. In absence of a finite element analysis model suitable for the maxillary ANS region, cortical bone stress under different insertion angles should be investigated in the future. However, if the cortical bone stress is indeed maximal at the insertion angle of 30°, platform creation is required to reduce condensed stress and avoid slippage. The most qualified height level concentrated between 14 mm to 16 mm and 22 mm or 24 mm should be avoided due to the uncertainty of ANS region morphology. Pre-surgical CBCT or three-dimensional reconstruction should be assessed and evaluated concerning implant site thickness to avoid any potential fracture during operation.

When it comes to different insertion angles, the AUCs in Figure 4D presented no statistically significant difference under different insertion angles through one-way ANOVA analysis. Sex and growth pattern were statistically significantly associated with CBT, as established by the linear regression analysis in Table 2. A sexual dimorphic association was observed for CBT, with males having larger biting force and masticatory muscles than females [38]. Our results partially align with those of Ono et al. [39] who found that the cortical bone was thicker in males than in females at vertical levels of 5 to 9 mm apical to the alveolar crest in the maxilla. However, in other possible mini-screw insertion sites, such as the mandible, the same sexual dimorphic association was not observed [40]. Ozdemir et al. [31] observed that adults with a hypodivergent facial type tend to exhibit thicker CBT in the maxillary alveolar process. Our research is the first to reveal a thicker cortical bone of the maxillary basal bone and ANS region in hypodivergent patients. Increased facial divergence is associated with reduced muscle function, which plays a critical role in the remodeling and bony adaptation of the maxilla, resulting in different CBT [41,42]. When dealing with hyperdivergent patients, clinicians can increase the diameter of the mini-screw to prevent failure and ensure a sufficient CBT, and vigilance should be exercised to avoid fracture.

A recent study indicated that the length of the screw did not exhibit a statistically significant association with stability if the mini-implant was longer than 5 mm and recommended that the required diameter should be more than 1 mm [43]. Furthermore, considering the presence of ample soft tissue coverage in the ANS region, it recommended that the mini-implant should extend 3 mm beyond the trimmed bone surface to allow for passage through the mucosa. Therefore, based on these dimensional considerations, we propose utilizing a mini-implant with a diameter of 1.5 mm and length of 8 mm for placement in the ANS region.

Nevertheless, compared to the inter-radicular area, ANS insertion demands a higher level of surgical expertise from clinicians due to the thick soft tissue coverage, low frenum attachment, and prominent blade-shaped bone ridge. Previous clinical studies suggested that nonkeratinized mucosa was more susceptible to mini-implant insertion failure [44]. Additionally, the high insertion success rates observed in the attached gingiva and palatal area indicated that selecting the keratinized mucosa area as the insertion site would be beneficial [19]. Moreover, the blade-shaped bone ridge in the ANS would lead to slippage during mini-implant insertion. Therefore, relevant surgeries (e.g., flapping surgery, frenectomy, osteoplasty) should be performed prior to insertion in the ANS region, otherwise it may result in failure and potential complications thereafter.

Conventionally, the width of the attached gingiva is measured from the gingival margin to the mucogingival junction, and for maxillary incisors, this value was 3.9 mm (SD = 1.32 mm) according to an observational study conducted by Jennes ME et al. [45]. Chun, Y et al. [46] found that the distance between the tip of the inter-radicular papillae and the alveolar crest for maxillary anterior teeth ranged from 3.20 mm to 5.75 mm, and thus the tip of the interdental papillae could serve as a reliable visual guide for the positioning of mini-implants. However, the location of the interdental papillae has also been proven to be associated with the distance from the proximal contact to the alveolar bone crest [47]. The width of the attached gingiva is proposed to be measured from the interdental papillae between bilateral maxillary central incisors to the mucogingival junction, ranging from approximately 5.78 mm to 10.97 mm. The ANS start is included, implying that although it is anatomically high and deep, the mini-implant may still be inserted within the keratinized gingiva.

**Figure 5:**
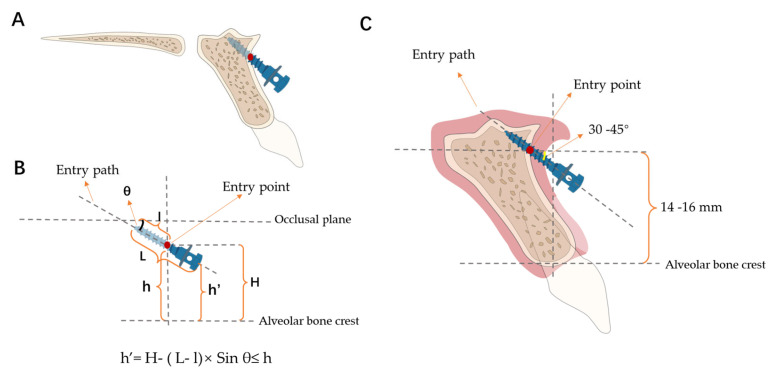
(**A**) Illustration of mini-implant insertion in the ANS region. (**B**) Illustration of the inequity in insertion height and angulation. H = entry point height, L = mini-implant length including the head, l = bone contact length, h = the height of mucogingival junction, h′ = the height of mini-implant head, and θ = insertion angle. (**C**) Illustration of the recommended mini-implant insertion height and angulation.

The head of the mini-implant posed challenges such as soft tissue encapsulation when placed in the nonkeratinized gingiva in clinical observations. Normally, it is best to insert the mini-screw inside the range of the attached gingiva to prevent any mucositis. However, the recommended insertion height is 14 to 16 mm, and the entry point may fall out of the height of the mucogingival junction. To address this, we proposed an innovative concept (Figure 5) to facilitate clinicians in optimizing the insertion angle and height parameters under given mini-implant dimensions. The theoretical foundation for which is that the height of the head of the mini-screw (h′) is required to be less than the height of the mucogingival junction (h). The height of the entry point (H) and insertion angle (θ) are correlated under a given height of the mucogingival junction, the fixed length of the mini-screw (L), and its intra-bony proportion (l). When either the insertion height level or angulation is limited, the other index would be pinned correspondingly. Under scenarios such as when the ideal insertion height is compromised or not achievable, adjustments in angulation could be made to satisfy the requirement. For example, when the ideal insertion height is way up to the nose, but the width of the attached gingiva is limited, the angulation should be increased to ensure the head of the mini-implant ends up in the range. With our mapping of the relationship between angulation and CBT and BD, optimal angulation for each specific height will be easily decided even when the insertion height is in the nonkeratinized area, thereby achieving stability and avoiding mucositis.

h′ = H − (L − l) × Sin θ ≤ h

(H = entry point height, L = mini-implant length including the head, l = bone contact length, h = the height of mucogingival junction, h′ = the height of mini-implant head, and θ = insertion angle).

This study is subject to certain limitations that should be acknowledged. Firstly, a retrospective design inherently carries limitations and potential biases. Additionally, the limited sample size may affect the generalizability of the findings in a broader population. Secondly, this study only explored limited influencing factors, potentially excluding others that could have had contributed to a more comprehensive understanding, such as morphology and phenotype classification in the ANS region, which future studies should include and a universal classification paradigm should be established. Thirdly, the accuracy of our results was contingent upon the precision and accuracy of the facility, specifically in regard to the localization of bone edges, relying solely on the accuracy of CBCT. Any limitations or inaccuracies in the CBCT imaging may have influenced the measurement of various bone-related indices. Future studies utilizing higher accuracy CBCT machines may yield more reliable and precise results.

## 5. Conclusions

In the ANS region, the dimensional requirements of mini-implants should be a diameter of 1.5 mm and length of 8 mm, and the recommended hard tissue parameter for insertion is a height level ranging from 14–16 mm from the alveolar bone crest to ensure a minimum of 3 mm CBT. Additionally, the recommended angulation for mini-implant insertion is between 30° and 45° with respect to the occlusal plane. Moreover, we proposed an inequity to optimize the insertion angulation under a given height to achieve better stability.

## Figures and Tables

**Table 1:** Cephalometric indexes of samples with different characteristics.

Characteristics	SNA	SNB	ANB	Wits Appraisal	GoGn-Sn
Age					
Adult (n = 27)	82.33 ± 2.71	80.24 ± 4.08	2.09 ± 3.67	−1.44 ± 5.08	32.92 ± 7.77
Adolescent (n = 23)	81.95 ± 2.86	80.29 ± 4.48	1.90 ± 3.63	−1.54 ± 4.94	32.69 ± 7.45
Gender					
Female (n = 27)	82.00 ± 2.91	79.81 ± 3.97	2.19 ± 3.49	−1.26 ± 4.90	32.98 ± 7.53
Male (n = 23)	81.95 ± 2.86	80.29 ± 4.48	1.90 ± 3.63	−1.54 ± 4.93	32.69 ± 7.45
Angle classification					
Class I (n = 14)	80.98 ± 3.65	78.45 ± 2.68	2.53 ± 1.86	−1.86 ± 3.00	34.56 ± 8.45
Class II (n = 20)	82.76 ± 2.76	77.91 ± 2.84	4.85 ± 1.85	2.11 ± 2.72	33.44 ± 6.86
Class III (n = 16)	81.79 ± 2.33	83.68 ± 4.67	−1.26 ± 3.20	−4.75 ± 5.32	30.82 ± 7.40
Growth pattern					
Hyperdivergent (n = 17)	82.17 ± 2.80	79.73 ± 4.03	2.44 ± 3.66	−1.23 ± 5.21	33.21 ± 7.93
Normodivergent (n = 21)	82.00 ± 2.95	79.76 ± 4.01	2.24 ± 3.52	−1.25 ± 4.97	32.12 ± 7.58
Hypodivergent (n = 12)	82.25 ± 2.72	80.58 ± 4.54	1.94 ± 3.74	−1.59 ± 5010	31.76 ± 7.73

**Table 2:** Results of mixed effects analysis for all measured data.

Factor	Bone Depth	Cortical Bone Thickness
Height	<0.0001	0.0036
Angle	<0.0001	0.0001
Height × Angle	<0.0001	<0.0001

**Table 3:** Linear regression analysis of multiple variables for bone depth, cortical bone thickness, and ANS start at 0° insertion angle.

Variables	Bone Depth		Cortical Bone Thickness		ANS Start	
	β (95% CI)	*p*-value	β (95% CI)	*p*-value	β (95% CI)	*p*-value
Age						
Adolescent	Referent					
Adult	0.056 (−0.697, 1.043)	0.69	−0.226 (−0.865, 0.073)	0.096	0.216 (−0.251, 2.137)	0.119
Gender						
Female	Referent					
Male	0.253 (−0.100, 1.666)	0.081	0.336 (0.111, 1.064)	0.017	−0.086 (−1.587, 0.838)	0.537
Growth pattern						
Hyperdivergent	−0.163 (−1.688, 0.509)	0.286	0.258 (−0.065, 1.121)	0.08	−0.024 (−1.629, 1.389)	0.873
Normodivergent	Referent					
Hypodivergent	0.241(−0.284, 1.676)	0.159	0.301 (0.026, 1.083)	0.04	−0.413 (−3.241, −0.550)	0.007

## Data Availability

The data presented in this study are available upon request from the corresponding author upon reasonable request.
